# Pink1 deficiency enhances neurological deficits and inflammatory responses after intracerebral hemorrhage in mice

**DOI:** 10.1016/j.neurot.2024.e00317

**Published:** 2024-01-23

**Authors:** Jingchen Li, Jianliang Li, Erkun Guo, Yuanyu Wang, Ming Yang, Haoran Huo, Yunpeng Shi, Lin Zhao

**Affiliations:** Department of Neurosurgery, The Second Hospital of Hebei Medical University, No. 215 Hepingxi Road, Shijiazhuang 050000, Hebei, China

**Keywords:** Pink1, Inflammation, Neurological deficits, Intracerebral hemorrhage

## Abstract

Pink1 (PTEN-induced putative kinase 1) is a protein associated with maintaining mitochondrial function and integrity and has been reported to mediate neurodegeneration and neuroinflammation. While the role of Pink1 in intracerebral hemorrhage (ICH)-related neurological deficits and inflammatory responses is not deciphered. Congenic blood was transfused into the left corpus striatum to construct the ICH model in C57/BL6 wild-type (WT) and Pink1^−/−^ mice. The relative expression of Pink1, monocyte chemoattractant protein-1 (MCP-1), macrophage inflammatory protein (MIP)-2, tumor necrosis factor (TNF)-α, interleukin (IL)-1β, Cd86, nitric oxide synthase 2 (Nos2), Cd206, arginase 1 (Arg-1), and IL-10 was detected with qRT-PCR, Western blotting, or ELISA. Mouse neurological deficit scores (mNSS) and water content were detected, and an open-field test was performed to assay anxiety-like behavior. Remarkably decreased Pink1 expression and increased MIP-2, IL-1β, MCP-1, and TNF-α expression were observed after 12 ​h, 24 ​h, 48 ​h, 72 ​h, and 7 ​d post-ICH induction in the ipsilateral injury hemispheres. Pink1 deficiency could further up-regulate mNSS scores, brain water content, MIP-2, MCP-1, IL-1β, and TNF-α in the ipsilateral injury hemispheres. On the other hand, Pink1 deficiency could decrease the number of center cross, the velocity, and the total distance traveled in open field test. Pink1 deficiency could further up-regulate the mRNA levels of pro-inflammatory (M1) molecules (Cd86, Nos2), and down-regulate the relative expression of anti-inflammatory (M2) molecules (Cd206, Arg-1, and IL-10). Pink1 deficiency deteriorates neurological deficits and inflammatory responses after ICH, which can be considered as a treatment target.

## Introduction

As the most devastating type of stroke, intracerebral hemorrhage (ICH) occurs at any age [1,2]. The absorption and expansion of the resulting hematoma can trigger a series of reactions, which lead to primary and secondary brain injury [3]. Mechanically, hematoma components may activate microglia and promote pro-inflammatory cytokines release to attract peripheral immune cell infiltration [4]. Depending on the location, ICH-affected individuals may suffer from the abrupt onset of headache, nausea, partial paralysis, personality changes, or cognitive abilities alteration [5]. In severe cases, ICH can cause permanent brain damage or death [6]. Unfortunately, no clinically proven therapies are available, and the primary treatment is only supportive [7].

Increasing evidence demonstrates that innate inflammatory reactions might participate in short-term toxicity, neurological deficits, and long-term recovery [8,9]. Considerable research has been performed to decipher the subsequent inflammatory cascade and to screen the potential therapeutic strategies [[10], [11], [12]]. Activated and polarized microglia, either pro-inflammatory or anti-inflammatory phenotypes, are identified to be critical in neural repair and inflammation inhibition processes [13].

Our previous investigation demonstrates that PTEN-induced putative kinase 1 (Pink1) promotes microglia mitochondrial autophagy to protect against ICH-induced brain injury [14]. In order to further study the significance of Pink1 in ICH and its relationship with ICH-induced neurological dysfunction and neuro-inflammation, time course neuro-inflammation molecule expression is detected. Pink1 deficiency mice are utilized to confirm the neurological protection and inflammatory inhibition role of Pink1 in ICH.

## Methods & Materials

### ICH model

C57BL/6J wild-type and C57BL/6J-Pink1^em1Cya^ (KOCMP-68943-Pink1-B6J-VA, Pink1^−/−^) mice purchased from Cyagen Saiye Biology (eight-week-old; male) were cultured in pathogen-free condition. Mice were anesthetized with intraperitoneal pentobarbital at a dose of 50 ​mg/kg and immobilized on a Model 1430 stereotaxic frame (Kopf Instruments). A stainless steel cannula (26-g) was used to transfuse congenic tail vein blood into the left corpus striatum with a burr hole (1 ​mm diameter) on the skull at the speed of 15 ​μL/5 ​min. In the sham group, a burr hole on the skull was drilled without congenic tail vein blood transfusion. The timeline of tissue collection (Pre-surgery, 12 ​h, 24 ​h, 48 ​h, 72 ​h, and 7 ​d post ICH; n ​= ​26) to detect the relative expression and neurological deficit scores detection (Pre-surgery, 1 ​d, 3 ​d, 7 ​d, and 14 ​d post ICH; n ​= ​8) were indicated in the supplementary Figure S1. Animal studies were approved by the ethics committee of the Second Hospital of Hebei Medical University.

### Reverse transcription-polymerase chain reaction (RT-PCR)

Total RNA extracted from ipsilateral injury hemispheres derived from different groups of mice with RNeasy Mini Kit (Qiagen, Valencia, CA, USA) was reverse-transcribed into complementary DNA with a High Capacity RNA-to-cDNA (Applied Biosystems, Waltham, MA). RT-PCR was performed with SYBR Green master mix (Roche, Penzberg, Upper Bavaria, Germany): 96 ​°C for 10 ​min, 45 cycles of 95 ​°C for 15 ​s, and 60 ​°C for 1 ​min. Primers used in this study: *Pink1*, F: 5′-TTCTTCCGCCAGTCGGTAG-3′, R: 5′-CTGCTTCTCCTCGATCAGCC-3’; *Cd86*, F: 5′-TCAATGGGACTGCATATCTGCC-3′, R: 5′-GCCAAAATACTACCAGCTCACT-3’; *Nos2*, F: 5′-GTTCTCAGCCCAACAATACAAGA-3′, R: 5′-GTGGACGGGTCGATGTCAC-3’; *Mcp-1*, F: 5′-TAAAAACCTGGATCGGAACCAAA-3′, R: 5′-GCATTAGCTTCAGATTTACGGGT-3’; *Arg-1*, F: 5′-GACCTGGCCTTTGTTGATGT-3′, R: 5′-CCATTCTTCTGGACCTCTGC-3’; *Cd206*, F: 5′-GGGACTCTGGATTGGACTCA-3′, R: 5′-GCTCTTTCCAGGCTCTGATG-3’; *Il-10*, F: 5′-CCAGTTTTACCTGGTAGAAGTGATG-3′, R: 5′-TGTCTAGGTCCTGGAGTCCAGCAGACTCAA-3’; *Iba-1*, F: 5′- CTTGAAGCGAATGCTGGAGAA-3′, R: 5′-AATTCGCCGGAGACACTCG-3’; *Gapdh*, F: 5′-AATGGATTTGGACGCATTGGT-3′, R: 5′-TTTGCACTGGTACGTGTTGAT-3’. *Gapdh* was utilized as internal control with the 2^−ΔΔCT^ method.

### Enzyme-linked immunosorbent assay (ELISA)

The relative content of IL-1β (BMS6002), MIP-2 (900-K152), MCP-1 (BMS6005), and TNF-α (88-7324-88) in the ipsilateral injury hemispheres was detected with relevant ELISA kits (eBioscience, San Diego, CA) according to the manufacturer's protocol. Optical density was measured with a SpectraMax M5 microplate reader (Molecular Devices, Sunnyvale, CA), and the wavelength was chosen as 450 ​nm.

### Western blotting

Ipsilateral injury hemispheres were isolated and lysed with Cell Lysis Buffer (Beyotime Biotechnology, Shanghai, China), and the soluble supernatants (20 ​μg) were loaded on 10 ​% sodium dodecyl sulfate–polyacrylamide gel electrophoresis and transferred to polyvinylidene fluoride membranes. After blocking with 5 ​% dry milk, the membranes were incubated with the primary antibody against Pink1 (sc-517353, Santa Cruz, Dallas, TX) at a 1:500 dilution and cleaved caspase 3 (ab214430, Abcam) at a 1:1000 dilution at 4 ​°C overnight and peroxidase-conjugated secondary antibody (Santa Cruz) at a 1:2000 dilution for 2 ​h, which was developed with a GE Healthcare ECL system (Tanon). GAPDH (1:1000, sc-365062, Santa Cruz) was utilized as an internal control.

### Water content

The degree of brain edema was revealed by the water content with wet/dry method. After the animals were anesthetized, the brain tissues were taken out immediately, and thick brain slices (4 ​mm) were cut around the injection marks, which were weighed with a Sartorius electronic balance as wet weight (WW). The weighed slides were further roasted at 100 ​°C for 24 ​h and weighed as dry weight (DW). The formula of [WW ​− ​DW]/WW ​× ​100 ​% was calculated as the water content of brain tissue (%).

### Neurological severity scores

The modified mouse neurological severity score (mNSS), including sensory, reflex, balance, and motor tests, was determined as previously described [15,16]. The severity was determined as follows: 0: without dysfunctions; 1–6: mild damage; 7–12: moderate damage; and 13–18: severe damage.

### Open field test

Locomotion activity was assayed in a light-grey polyvinyl chloride activity chamber (55 ​× ​55 ​× ​36 ​cm), which was divided into four quadrants. After placing mice in the center of the chamber, locomotion activity was recorded for 5 ​min (n ​= ​12 per group). The total time in the center zone, the frequency of the center cross, time spent near the walls, and outside the center were measured. Distance traveled, velocity, and reduced frequency of center cross were utilized as indications of anxiety behavior or locomotor activity.

### Statistical analysis

Data was shown with mean ​± ​standard deviation (SD). One-way ANOVA followed Dunn's multiple comparisons test or two-way ANOVA followed Tukey's multiple comparisons test was performed. Image J was utilized for quantitative analysis. The significance level was set as *p* ​< ​0.05 for both ANOVA and Spearman's correlation analysis.

## Results

### Pink1 negatively correlates with inflammatory cytokine expression

**Fig. 1:**
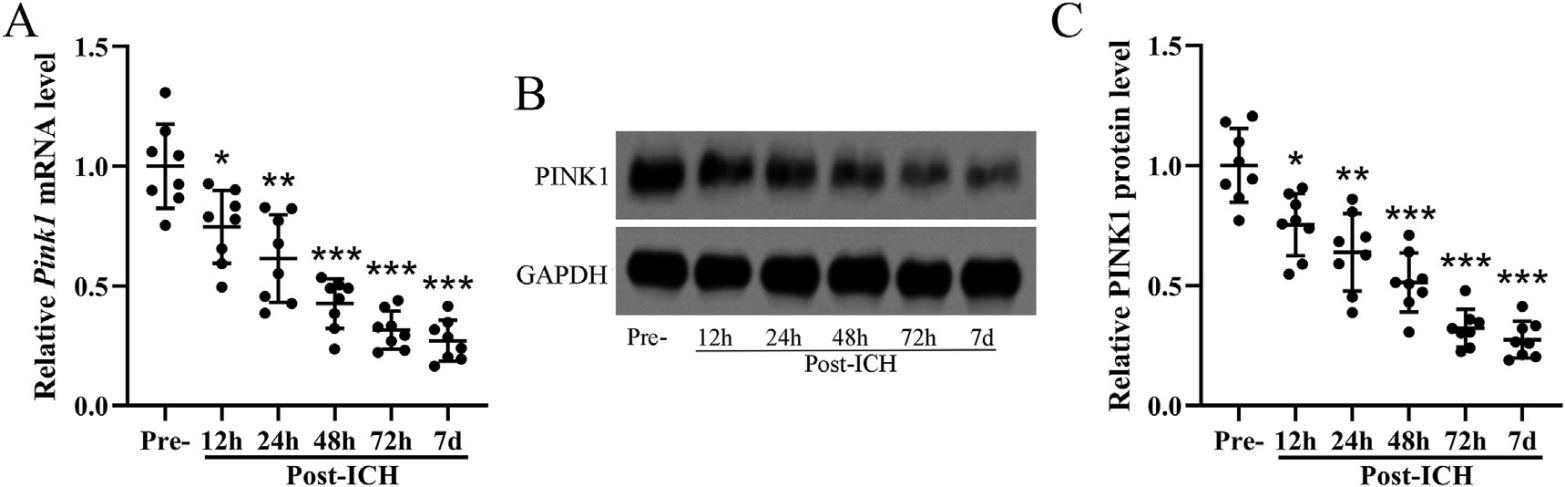
**Down-regulated Pink1 expression after intracerebral hemorrhage induction.** The relative mRNA (A) and protein (B and C) levels of Pink1 were detected with qRT-PCR and Western blotting in ICH mice ipsilateral injury hemispheres. The time point of Pre-surgery was used as control. Image J was utilized for quantitative analysis, and expression was normalized to control (the time point of Pre-). The data was shown with mean ​± ​SD. n ​= ​8 for each time point. ∗*p* ​< ​0.05, ∗∗*p* ​< ​0.01, ∗∗∗*p* ​< ​0.001 compared to control. One-way ANOVA followed Dunn's multiple comparisons test.

Time course analysis was utilized to reveal the expression of Pink1 after congenic blood infusion in the hemispheres. The relative mRNA (Fig. 1A) and protein (Fig. 1B and C) levels of Pink1 in the ipsilateral injury hemispheres of wild-type mice were detected, as expected, remarkably decreased Pink1 expression was observed after 12 ​h, 24 ​h, 48 ​h, 72 ​h, and 7 ​d post ICH. It was worth noting that the relative expression of Pink1 demonstrated time-dependent decrease trends at the acute phase (0–72 ​h), while on 7 ​d, a plateau occurred.

**Fig. 2:**
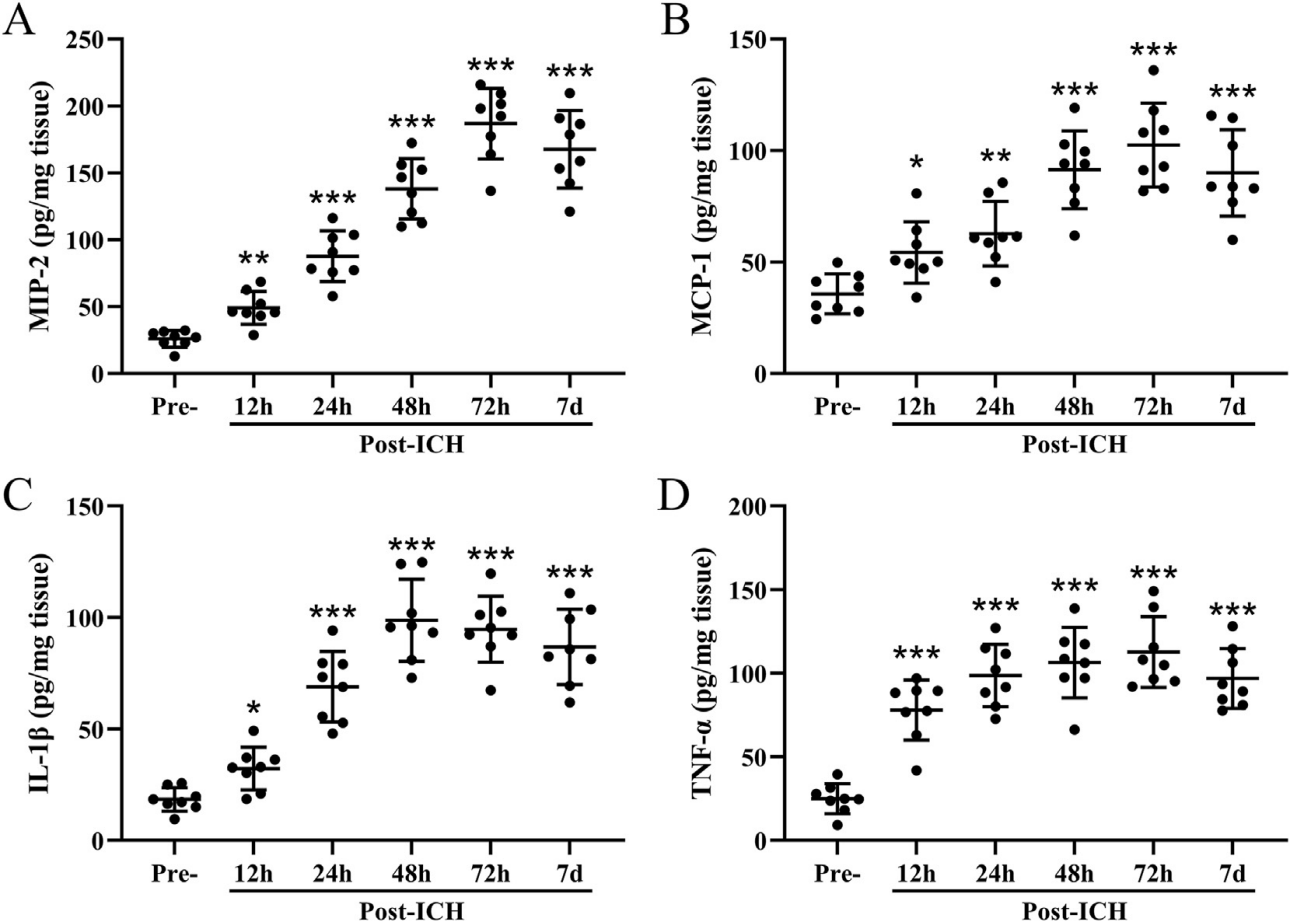
**Intracerebral hemorrhage induced inflammatory responses in ipsilateral injury hemispheres.** The levels of MIP-2 (A), MCP-1 (B), IL-1β (C), and TNF-α (D) in the ipsilateral injury hemispheres were examined by ELISA. The data was shown with mean ​± ​SD. n ​= ​8 for each time point. ∗*p* ​< ​0.05, ∗∗*p* ​< ​0.01, ∗∗∗*p* ​< ​0.001 compared to control. One-way ANOVA followed Dunn's multiple comparisons test.

On the other hand, up-regulated MIP-2 (Fig. 2A), MCP-1 (Fig. 2B), IL-1β (Fig. 2C), and TNF-α (Fig. 2D) in the ipsilateral injury hemispheres were revealed by ELISA assay after 12 ​h, 24 ​h, 48 ​h, 72 ​h, and 7 ​d post ICH. Our observation also indicated time-dependent increase trends of MCP-1, MIP-2, IL-1β, and TNF-α at the acute phase (0–72 ​h), while on 7 ​d, a plateau also occurred.

**Fig. 3:**
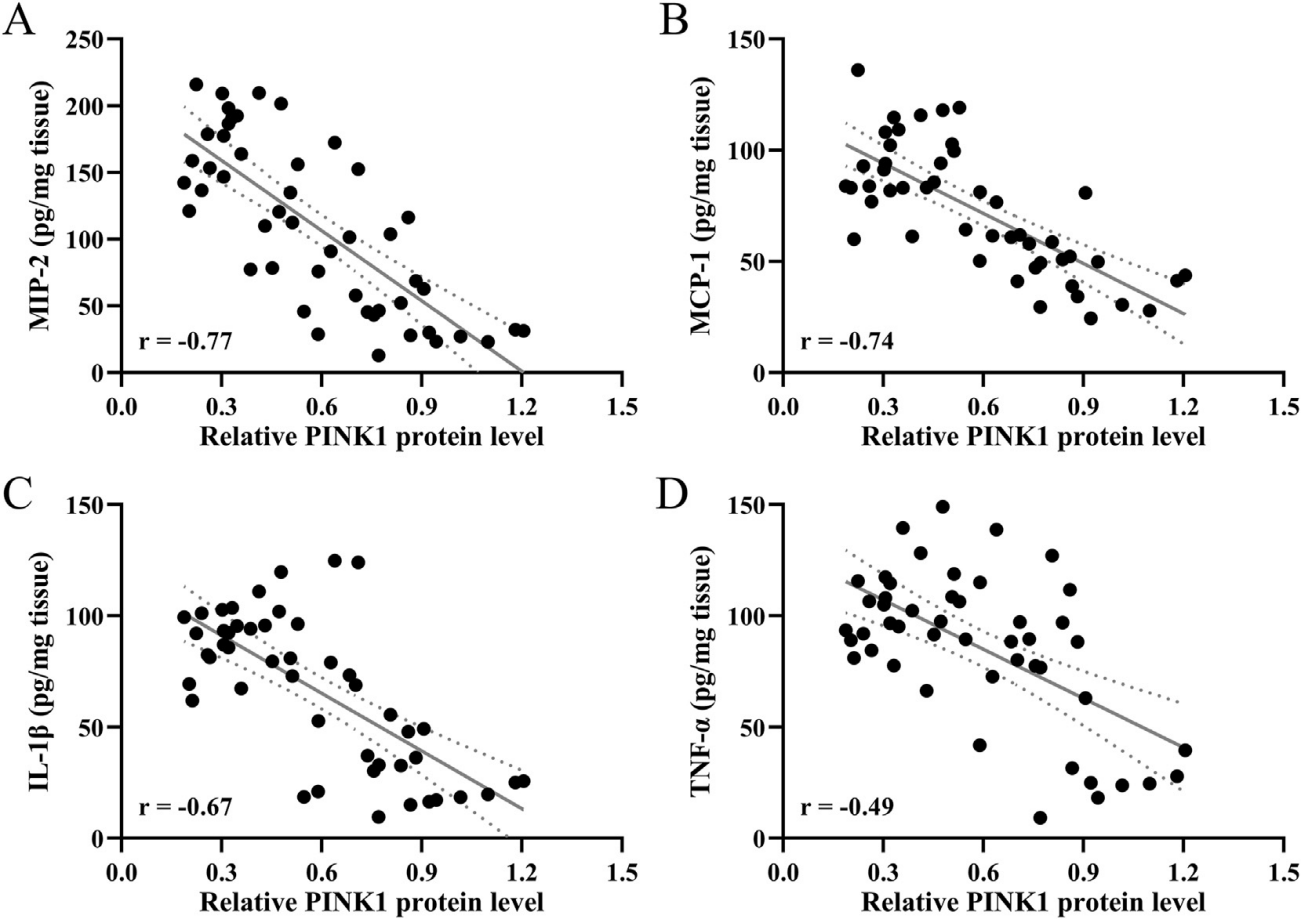
**The correlation between Pink1 and inflammatory cytokines.** Spearman's correlation between Pink1 and MIP-2 (A), MCP-1 (B), IL-1β (C), and TNF-α (D) in ICH-induced wild-type mice ipsilateral injury hemispheres. *p* ​< ​0.001 in all the analyses.

Spearman's correlation analysis between Pink1 and MIP-2 (Fig. 3A, *r* ​= ​−0.77), MCP-1 (Fig. 3B, *r* ​= ​−0.74), IL-1β (Fig. 3C, *r* ​= ​−0.67), and TNF-α (Fig. 3D, *r* ​= ​−0.49) demonstrated a significant negative correlation.

### Pink1 deficiency enhances neurological deficits and brain edema

**Fig. 4:**
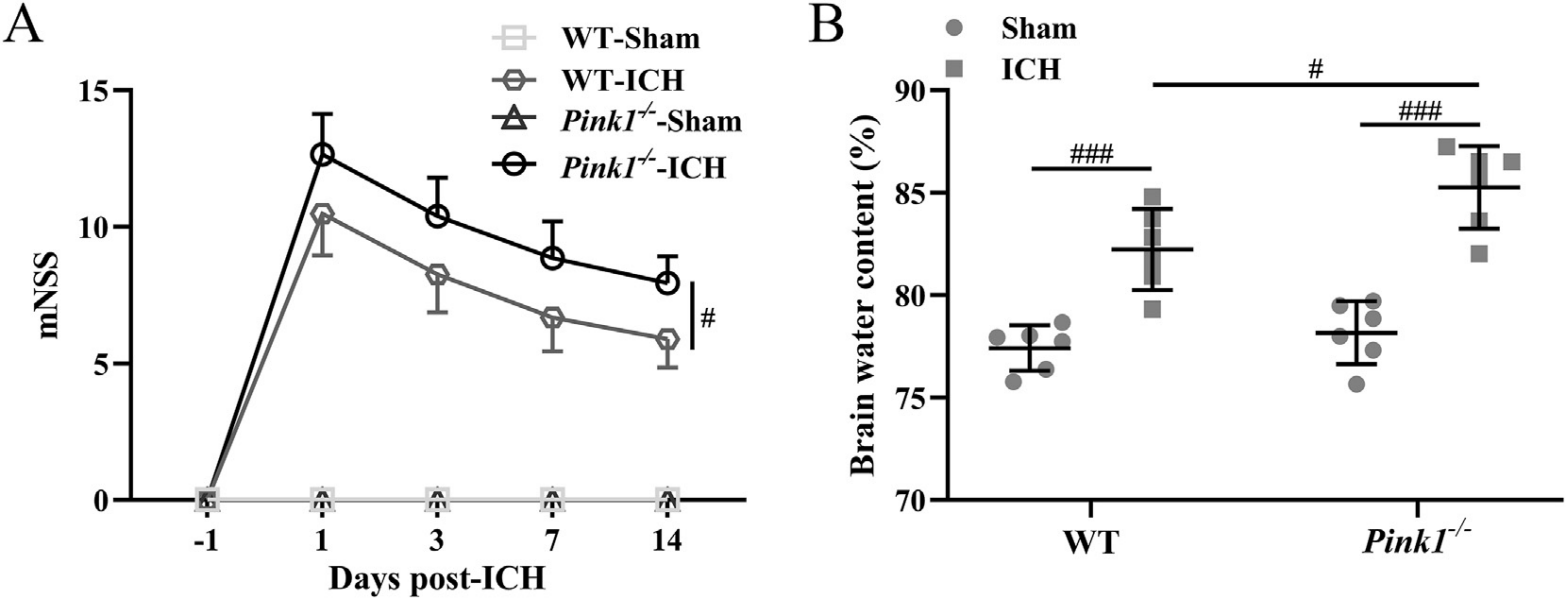
**Pink1 deficiency enhanced brain edema and neurological deficits induced by ICH.** Mouse neurological deficit scores (mNSS) were measured pre, 1, 3, 7, and 14 days post-ICH (A). n ​= ​10 for each group. Brain water content in the ipsilateral injury hemispheres was detected three days post-ICH (B). n ​= ​6 for each group. The data was shown with mean ​± ​SD. #*p* ​< ​0.05, ###*p* ​< ​0.001 from Two-way ANOVA followed Tukey's multiple comparisons test.

The phenotype of Pink1 wide-type mice and Pink1 deficiency mice was confirmed by Western blotting detection (supplementary Figure S2). On 1, 3, 7, and 14 days after ICH induction, mNSS scores peaked on day 1, and then decreased in both Pink1 wide-type mice and Pink1 deficiency mice (Fig. 4A). At the same time, increased brain water content was measured 3 days after ICH induction in both wide-type mice and Pink1 deficiency mice (Fig. 4B). All of these indicated the success of ICH model construction. On the other hand, Pink1 deficiency further up-regulated mNSS scores (Fig. 4A, *p* ​< ​0.05), brain water content (Fig. 4B, *p* ​< ​0.05), and the relative expression of cleaved Caspase 3 (supplementary Figure S3A and S3B). These results testified that Pink1 deficiency enhanced brain edema, neurological deficits, and apoptosis caused by ICH.

### Pink1 deficiency promotes anxiety-like behavior

**Fig. 5:**
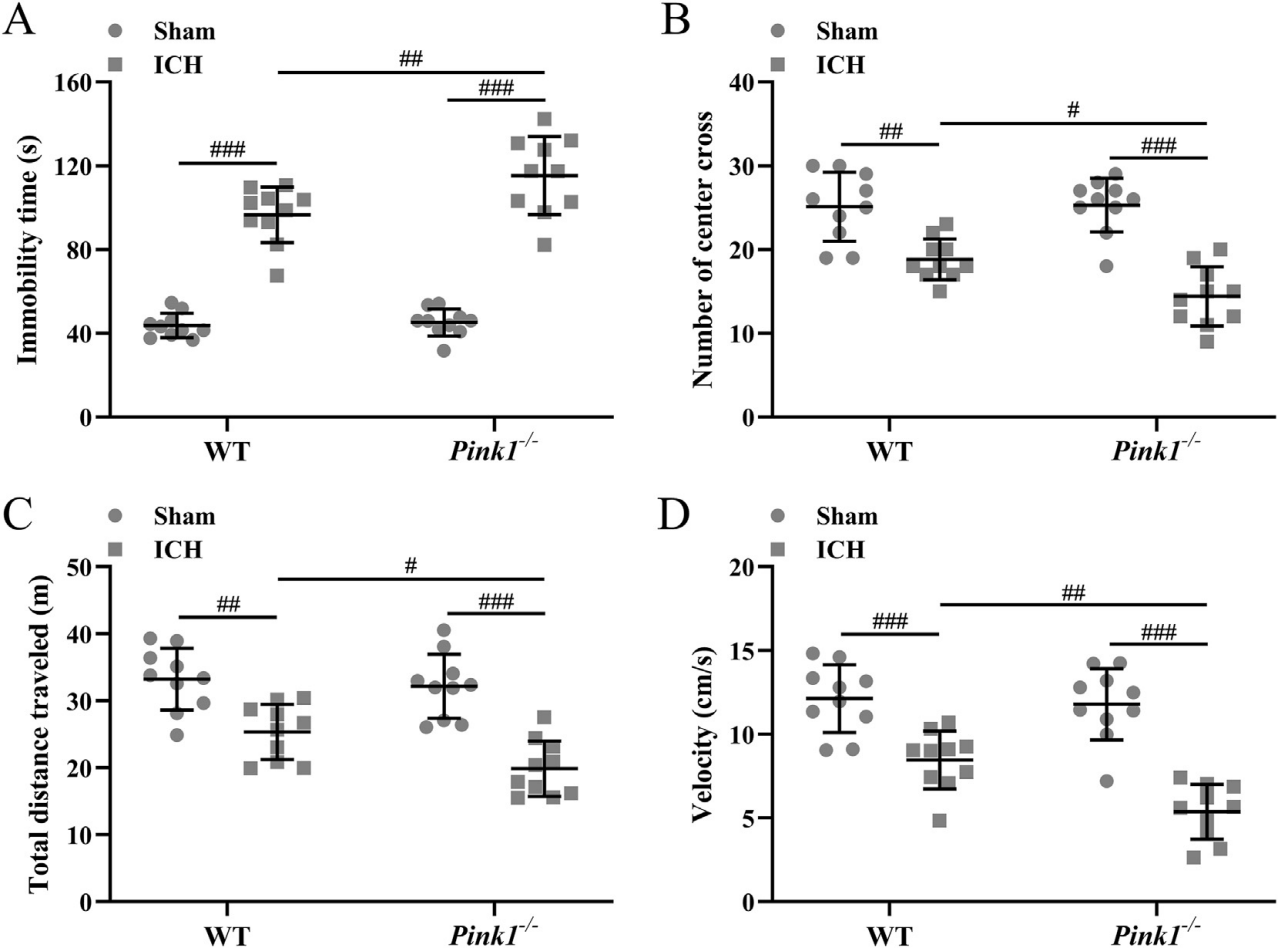
**Pink1 deficiency enhanced anxiety-like behavior induced by intracerebral hemorrhage.** Pink deficiency increased the immobility time (A), as well as decreased the number of center crosses (B), the total distance traveled (C), and the velocity (D). n ​= ​10 for each group. The data was shown with mean ​± ​SD. #*p* ​< ​0.05, ##*p* ​< ​0.01, ###*p* ​< ​0.001 from Two-way ANOVA followed Tukey's multiple comparisons test.

ICH induction could increase immobility time (Fig. 5A), as well as decrease the number of the center cross (Fig. 5B), the total distance traveled (Fig. 5C), and the velocity (Fig. 5D) in both wide-type and Pink1 deficient mice. On the other hand, Pink1 deficiency further increased the immobility time (Fig. 5A, *p* ​< ​0.01) and decreased the number of center cross (Fig. 5B, *p* ​< ​0.05), the total distance traveled (Fig. 5C, *p* ​< ​0.05), and the velocity (Fig. 5D, *p* ​< ​0.01). All of these data indicated that Pink1 deficiency could promote the anxiety of mice caused by ICH.

### Pink1 deficiency up-regulates ICH-induced inflammatory responses

**Fig. 6:**
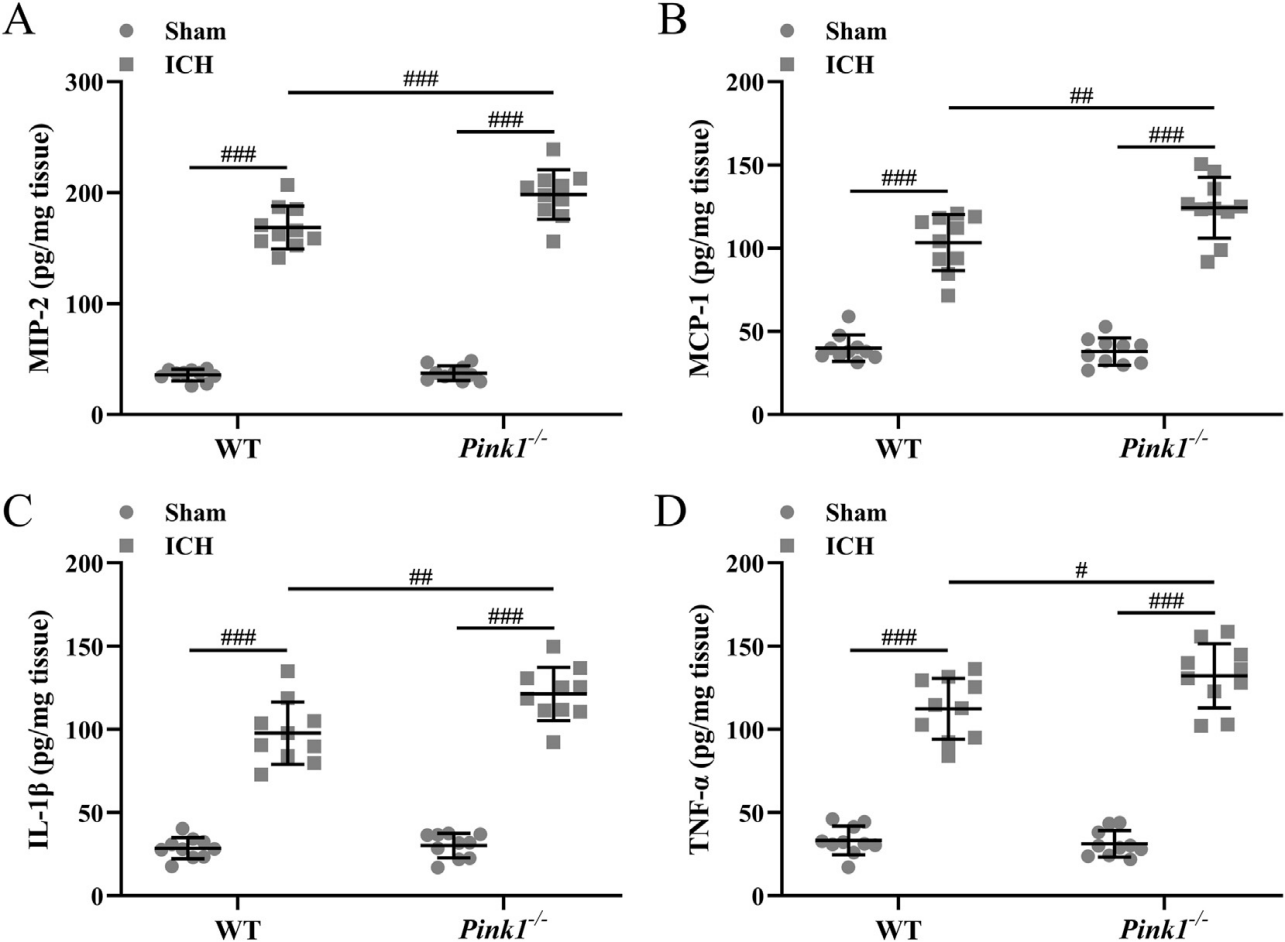
**Pink1 deficiency enhanced intracerebral hemorrhage-induced inflammatory responses.** The levels of MIP-2 (A), MCP-1 (B), IL-1β (C), and TNF-α (D) in the ipsilateral injury hemispheres were examined by ELISA at three days post-ICH. n ​= ​10 for each group. The data was shown with mean ​± ​SD. #*p* ​< ​0.05, ##*p* ​< ​0.01, ###*p* ​< ​0.001 from Two-way ANOVA followed Tukey's multiple comparisons test.

In wide-type mice, ICH induction up-regulated the levels of MIP-2 (Fig. 6A), MCP-1 (Fig. 6B), IL-1β (Fig. 6C), and TNF-α (Fig. 6D) in the ipsilateral injury hemispheres of both wide-type mice and Pink1 deficient mice. On the other hand, Pink1 deficiency further up-regulated the levels of MIP-2 (Fig. 6A, *p* ​< ​0.001), MCP-1 (Fig. 6B, *p* ​< ​0.01), IL-1β (Fig. 6C, *p* ​< ​0.01), and TNF-α (Fig. 6D, *p* ​< ​0.05) in the ipsilateral injury hemispheres. These data demonstrated that Pink1 deficiency could aggravate ICH-induced inflammatory responses.

### Pink1 deficiency increases pro-inflammatory cytokine and reduces anti-inflammatory cytokine expression in the ipsilateral injury hemispheres

**Fig. 7:**
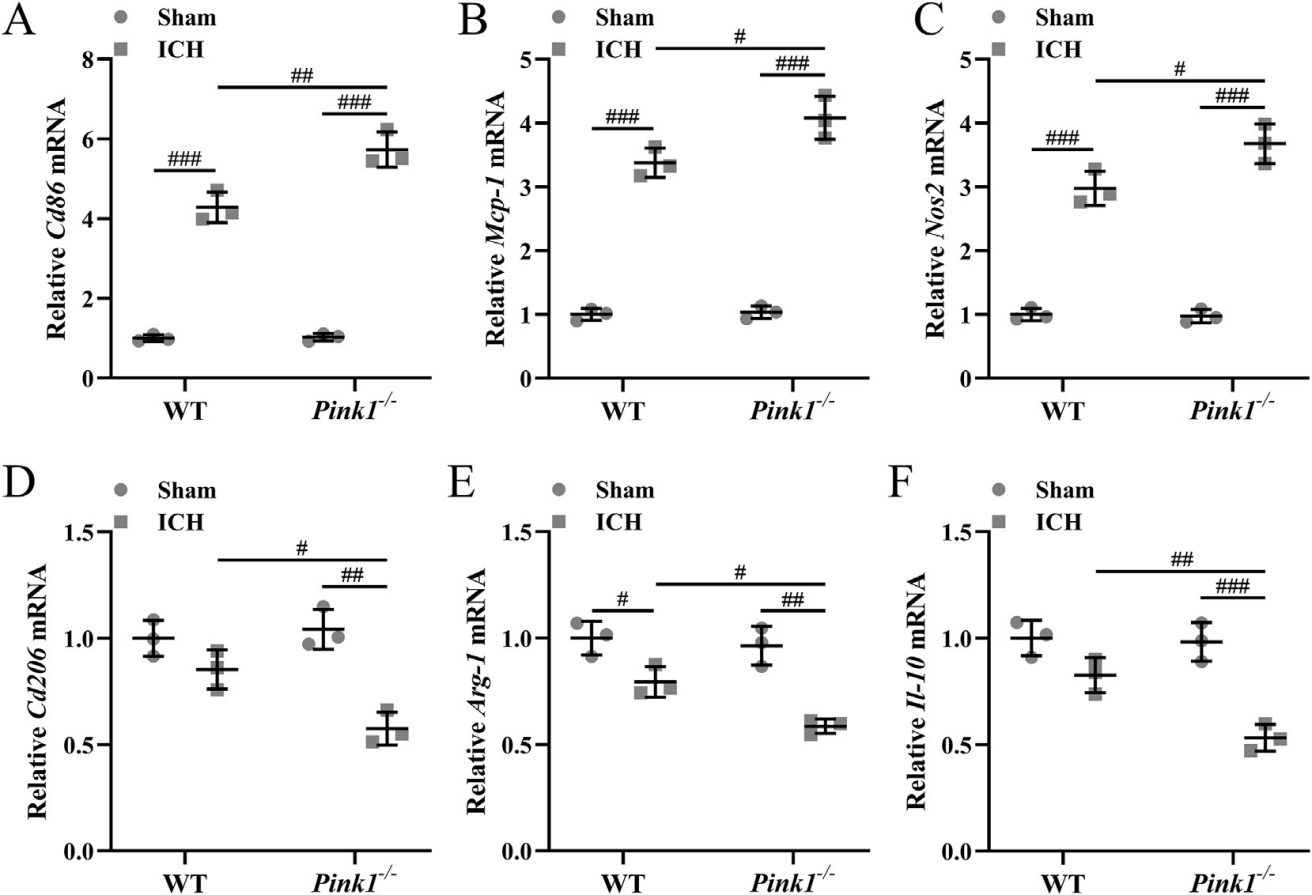
**Pink1 deficiency enhanced microglia M1-type polarization but inhibited M2-type polarization at three days post-ICH in the ipsilateral injury hemispheres.** The relative expression of Cd86 (A), Mcp-1 (B), Nos2 (C), Cd206 (D), Arg-1 (E), and Il-10 (F) in the ipsilateral injury hemispheres. n ​= ​3 repeats for each group (10 tissue homogenates were mixed for each group). The data was shown with mean ​± ​SD. #*p* ​< ​0.05, ##*p* ​< ​0.01, ###*p* ​< ​0.001 from Two-way ANOVA followed Tukey's multiple comparisons test.

Pro-inflammation (M1) associated genes (*Nos2*, *Cd86*) and anti-inflammation (M2) associated genes (*Arg-1*, *Cd206*, and *Il10*) were also detected. ICH up-regulated the mRNA levels of *Cd86* (Fig. 7A), *Mcp-1* (Fig. 7B), *Nos2* (Fig. 7C) and down-regulated the relative expression of *Arg-1* (Fig. 7E) in the ipsilateral injury hemispheres of both wide-type mice and Pink1 deficient mice. At the same time, Pink1 deficiency further up-regulated the mRNA levels of *Cd86* (Fig. 7A, *p* ​< ​0.01), *Mcp-1* (Fig. 7B, *p* ​< ​0.05), *Nos2* (Fig. 7C, *p* ​< ​0.05), and down-regulated the relative expression of *Cd206* (Fig. 7D, *p* ​< ​0.05), *Arg-1* (Fig. 7E, *p* ​< ​0.05), and *Il-10* (Fig. 7F, *p* ​< ​0.01). On the other hand, the relative time course (12 ​h, 24 ​h, 48 ​h, 72 ​h, and 7 ​d post-ICH) expression of Iba1, cd86, and cd206 was also detected. Up-regulated Iba1 (supplementary Figure S4A) and cd86 (supplementary Figure S4B) expression was observed at 12, 24, 48, and 72 ​h after ICH induction when compared with pre-surgery stage, while down-regulated cd206 expression (supplementary Figure S4C) was observed at 48 and 72 ​h after ICH induction when compared with pre-surgery stage. Spearman's correlation analysis demonstrated that Iba1 (*r* ​= ​−0.57, supplementary Figure S4D) and Cd86 (*r* ​= ​−0.74, supplementary Figure S4E) showed negative correlation with Pink1, and Cd206 (*r* ​= ​−0.66, supplementary Figure S4F) showed positive correlation with Pink1. All of these indicated that Pink1 deficiency could affect inflammatory responses in the ipsilateral injury hemispheres of ICH mice.

## Discussion

Although significant progress has been made, ICH is still a serious public health problem in adults worldwide with neurological deficits and inflammatory responses. Our time course analysis confirms the time-dependent decrease of Pink1 expression in the ipsilateral injury hemispheres in ICH. Pink1 deficiency can enhance neurological deficits, brain edema, and anxiety-like behavior after ICH. Functionally, Pink1 deficiency can enhance ICH-induced inflammatory responses and alter pro-inflammation and anti-inflammation-associated gene expression. All of these studies indicate that Pink1 deficiency enhances inflammatory responses and neurological deficits after ICH.

In this investigation, we demonstrate that Pink1 deficiency could enhance inflammatory responses in the ipsilateral injury hemispheres. It must be mentioned that both central nervous system-intrinsic microglia and monocyte-derived macrophages might contribute to the development of ICH. Although the precise cellular source of pro-inflammation- and anti-inflammation-associated gene expression in ICH is not indicated, intrinsic microglia or monocyte-derived macrophages is assumed as the main cellular source.

As resident immune cells in the central nervous system, microglia not only remove the hematoma and clear debris but also make M1 and M2 phenotype change to produce pro-inflammatory cytokines and neuroprotective anti-inflammatory cytokines to determine the progression of ICH [13,[17], [18], [19]]. In LPS/IFN-γ stimulated inflammation, Pink1 deficiency can attenuate expression of both pro-inflammatory and anti-inflammatory cytokines in microglia, while enhancing pro-inflammatory phenotypes of mixed astrocytes/microglia and pure astrocytes [20]. To what extent, Pink1 deficiency enhanced neurological deficits and inflammatory responses after ICH can be attributed to resident microglia is an interesting research field.

In this study, the relative expression of MCP-1, one vital chemokine to regulate infiltration and migration of macrophages [21], is also significantly altered upon Pink1 deficiency. In a murine model of polymicrobial infection, Pink1-deficient macrophage shows a pronounced increase in Δψm, leading to the metabolic rewiring in macrophage activation [22]. Mechanically, Pink1 improves retinoic-acid-inducible gene I-like receptors (RLRs)-triggered type I interferon production in macrophages by inhibiting TNF receptor-associated factor degradation [23].

Some limitations should be acknowledged here. Although our previous analysis demonstrates that after ICH induction, down-regulated PINK1 expression mainly happened in microglia instead of astrocytes, oligodendrocytes, and neurons [24]. Condition or microglia-specific knockout mice should be utilized to decipher the specific role of Pink1 in microglia in this study. On the other hand, only the relative expression of pro- and anti-inflammatory associated molecules is detected in the ipsilateral injury hemispheres, and cellular phenotype analysis should be performed in the future. Sex dimorphism has been demonstrated to affect experimental intracerebral hemorrhage, and only male mice are chosen in this study. The potential function of Pink1 deficiency on female ICH mice should also be investigated in the future. Last, the detailed mechanisms of how Pink1 deficiency impact ICH remain unclear. For example, it is not clear through what types of cells Pink1 functions during ICH.

Our study testifies that Pink1 deficiency can enhance ICH-caused neurological deficits and inflammatory responses.

## Ethical approval

Animal studies were approved by the ethics committee of Second Hospital of Hebei Medical University. This study was performed in strict accordance with the NIH guidelines for the care and use of laboratory animals (NIH Publication No. 85-23 Rev. 1985).

## Funding

This study was supported by the 10.13039/501100003787Natural Science Foundation of Hebei Province (H2023206116, H2022206456), Hebei Medical Science Research (20240027).

## Author contribution

Jingchen Li, Jianliang Li, Erkun Guo, Yuanyu Wang, Ming Yang, Haoran Huo, Yunpeng Shi, Lin Zhao conducted the experiments, analyzed the data, wrote the manuscript. Lin Zhao supervised the study.

## Data availability statement

The raw data supporting the conclusions of this article will be made available on request to the corresponding author by email.

## Declaration of competing interest

The authors declare that they have no known competing financial interests or personal relationships that could have appeared to influence the work reported in this paper.
