# Supplementary material for: Pink1 deficiency enhances neurological deficits and inflammatory responses after intracerebral hemorrhage in mice
Source: Neurotherapeutics. 2024 Jan 23;21(2):e00317. doi: 10.1016/j.neurot.2024.e00317 (PMC10963940; doi:10.1016/j.neurot.2024.e00317)
Supplement: Multimedia component 1 [file mmc1.docx]

**Supplementary materials**


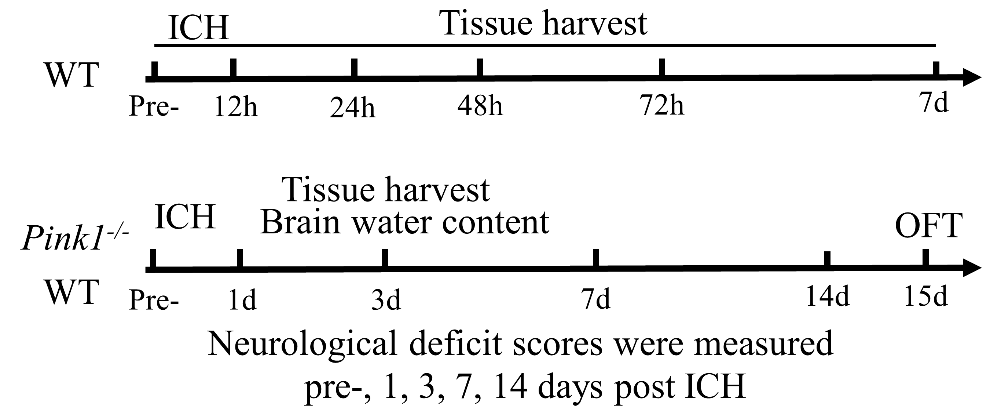


**Figure S1.** timeline of the experiments in this study.


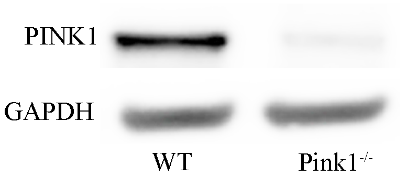


**Figure S2.** Protein expressions of Pink1 in the brain tissues from wild type and Pink1 deficiency mice.


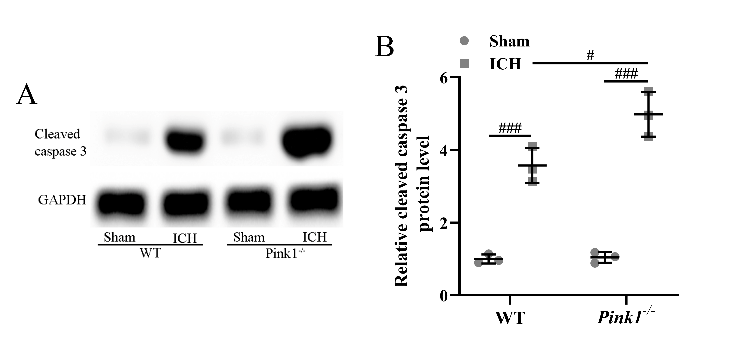


**Figure S3. Pink1 deficiency enhanced intracerebral hemorrhage-induced cell apoptosis.** The relative protein (A and B) levels of cleaved caspase 3 were detected with Western blotting in ICH mice ipsilateral injury hemispheres. n = 3 repeats for each group (10 tissue homogenates were mixed for each group). Date was shown with mean ± SD. #*p* < 0.05, ##*p* < 0.01, ###*p* < 0.001 from Two-way ANOVA followed Tukey's multiple comparisons test.


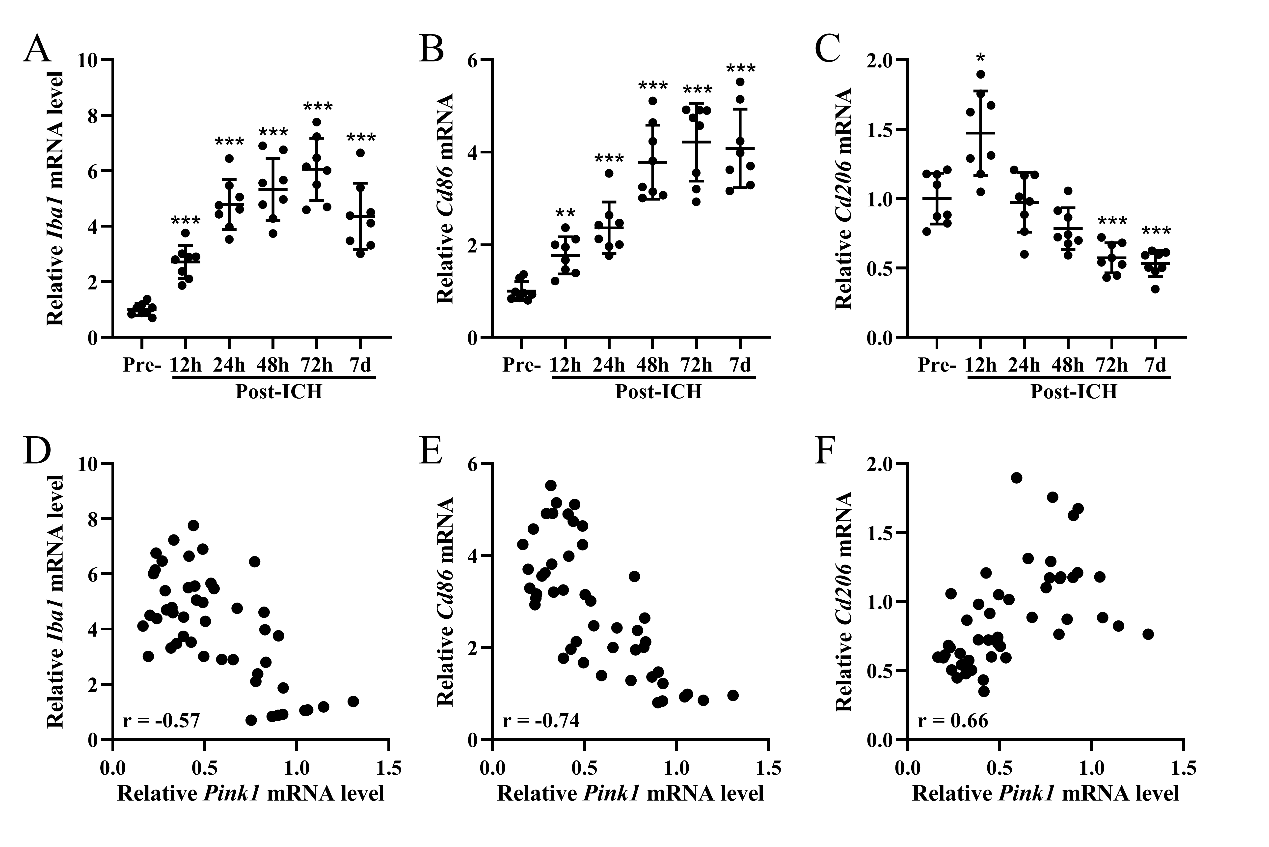


**Figure S4. Iba1 expressions and the correlation of Pink1 with M1/M2 polarization.** The relative mRNA levels of Iba1 (A), Cd86 (B) and Cd206 (C) in ICH mice ipsilateral injury hemispheres. The time point of Pre-surgery was used as control. Date was shown with mean ± SD. n = 8 for each time point. **p* < 0.05, ***p* < 0.01, ****p* < 0.001 compared to control. One-way ANOVA followed Dunn's multiple comparisons test. Spearman’s correlation between Pink1 mRNA expression and the mRNA expressions of Iba1 (D), Cd86 (E) and Cd206 (F) in ICH-induced wild-type mice ipsilateral injury hemispheres. *p* < 0.001.
